# Immune monitoring technology primer: immunoprofiling of antigen-stimulated blood

**DOI:** 10.1186/s40425-016-0122-4

**Published:** 2016-03-15

**Authors:** Laura Rosa Brunet, Samuel LaBrie, Thorsten Hagemann

**Affiliations:** Immodulon Therapeutics Ltd, Stockley Park 6-9 The Square, Uxbridge, UB11 1FW UK; Myriad RBM, 3300 Duval Road, Austin, TX 78759 USA

## Name(s) of the technology

TruCulture® Whole Blood Collection and Culture system is a whole blood collection and stimulation platform from Myriad RBM (Austin, USA).

## Description of the technology

Compelling evidence from several studies on the immune contexture of the tumor has revealed that infiltration by specific leukocyte cell subsets with specific cytokine signatures is linked with favorable outcomes in a variety of different cancers [1, 2]. Given the potential burden and limitation of repeat biopsies, there is a need for non-invasive techniques to complement the characterization of the immune contexture within the tumor and provide prognostic biomarkers for use in clinical immune monitoring. Advances in genomics, proteomics and metabolomics offer promise but have not yet translated to significant progress in this area. Nevertheless circulatory immunological biomarkers could potentially aid clinical decision-making regarding initiation, cessation, escalation or change of treatment and assessment of therapeutic responses. Moreover, in the context of immunotherapy, immunological biomarkers could serve as indicators of immunological competence and therefore susceptibility to immune-based therapies. As the success of immunotherapies is dependent on a functioning immune system and the development and/or restoration of anti-tumor adaptive immune responses, determining the level of the patient immune competency prior to or during immunotherapy may be critical. This assessment can be based on changes in secretion of both T-cell derived or APC-secreted cytokines and chemokines under specific stimulatory conditions and may ultimately help guide clinical choices.

**Fig. 1:**
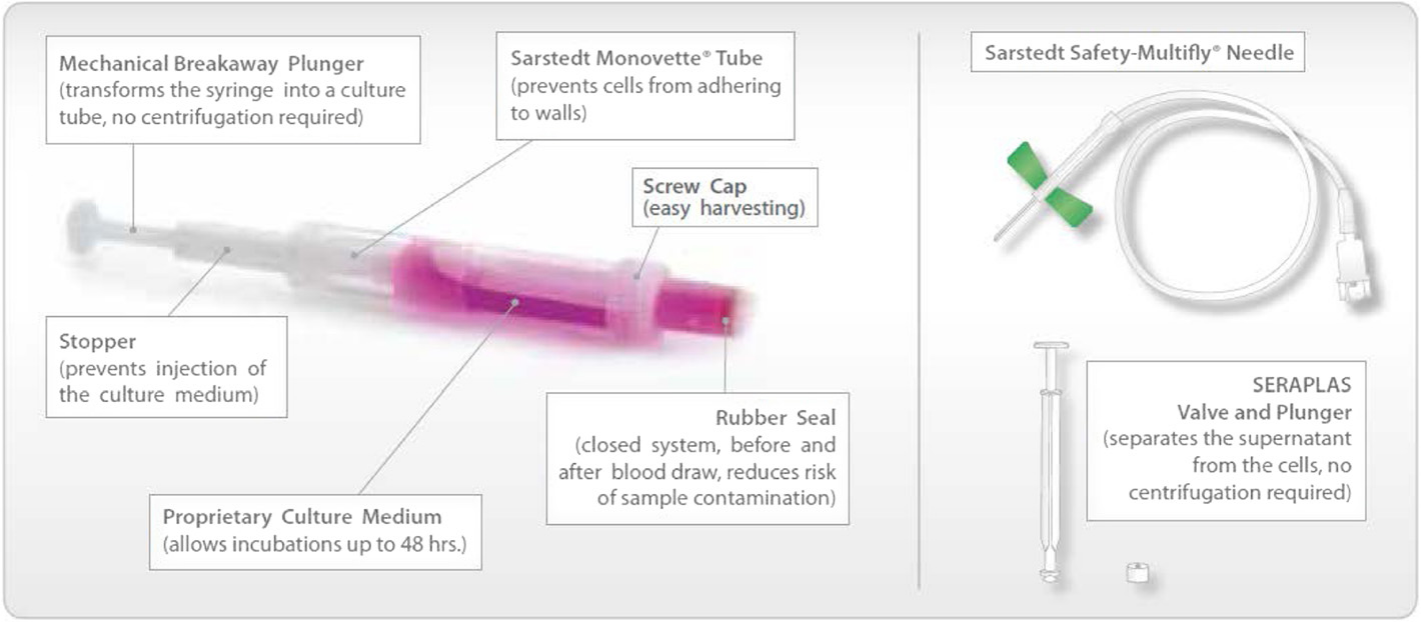
Workflow TruCulture® Whole Blood Collection and Culture system

The TruCulture® system is a syringe-based device designed for point of care use, allowing for sterile collection of whole blood (Fig. 1). The tube contains 1 ml of cell culture medium which may include a variety of immunological stimulants aimed at different cell subsets (Table 1). In these tubes, similar to blood collection tubes, 1 ml of whole blood is drawn in and mixed with the medium. Control tubes with no stimulants to assess background levels of mediators of interest are included. The tube is incubated at 37 °C in a dry heat-block for 24 to 48 h. After incubation, cells are separated by a valve separator component. The supernatant, now free of cells, is stored at −80 °C until analysis. This process does not require access to laboratory equipment such as sterile hood, CO_2_ incubator, centrifuge etc. or specialized training, so it has the potential to become a research tool easily integrated into clinical trial protocols and performed at point of care by nursing staff.

**Table 1:** Stimulants currently available for the TruCulture® Whole Blood Collection and Culture system

Description of stimulant	Target
Null TruCulture (TC) tube (TC Tubes Null)	Control
Tumor Necrosis factor (TNF)-alpha tube (TC Tubes TNF-α 10 ng/ml)	Cytokine
Intron A/Interferon (IFN)-alpha tube (TC Tubes IFN-α)	Cytokine
Interferon (IFN)-beta tube (TC Tubes IFN-β)	Cytokine
Interleukin (IL)-1beta + TNF-α tube (TC Tubes IL-1β + TNF-α)	Cytokine
Intron A/IFN-α + anti-CD3 + anti-CD28 tube (TC Tubes IFN-α + CD3 + CD28)	Cytokine
Intron A/IFN-α + Lipopolysaccharide (LPS)-EB (Escherichia coli 0111:B4) high concentration tube (TC Tubes IFN-α + LPS-EB)	Cytokine/TLR4 Ligand
Adenosine Triphosphate (ATP) + LPS-EB tube (TC Tubes ATP + LPS-EB)	NLRP3 Inflammasome/TLR4 Ligand
Lauroyl-γ-D-glutamyl-meso-diaminopimelic acid (C12-iE-DAP) tube (TC Tubes C12-iE-DAP)	NOD Ligand
Lipoarabinomannan (LAM) from *M. smegmatis* tube (TC Tubes LAM-MS)	NOD Ligand
Staphylococcal Enterotoxin B (SEB) tube (TC Tubes SEB)	T-Cell
Anti-CD3/CD28 tube (TC Tubes CD3 + CD28)	T-Cell
SEB/anti-CD28 tube (TC Tubes SEB + CD28)	T-Cell
anti- CD3 tube (TC Tubes CD3)	T-Cell
Zymosan (ZYM) tube (TC Tubes ZYM)	TLR2 Ligand
Fibroblast-stimulating Lipopeptide (FSL) tube (TC Tubes FSL-1)	TLR2 Ligand
Heat killed *E. coli* 0111:B4 tube (TC Tubes HKEB)	TLR2 Ligand
Heat Killed *Lactobacillus rhamnosus* tube (TC Tubes HKLR)	TLR2 Ligand
Polyinosinic:polycytidylic acid (Poly I:C) tube (TC Tubes Poly I:C)	TLR3 Ligand
LPS tube (TC Tubes LPS)	TLR4 Ligand
LPS-EB high concentration tube (TC Tubes LPS-EB)	TLR4 Ligand
LPS + SEB tube (TC Tubes LPS + SEB)	TLR4 Ligand/T-Cell
Resiquimod R848 tube (TC Tubes R848)	TLR7/8 Ligand
Gardiquimod (GDQ) tube (TC Tubes GDQ)	TLR7/8 Ligand
Class A CpG oligonucleotide + LPS-EB high concentration tube (TC Tubes ODN2216 + LPS-EB)	TLR9 Ligand

This technique may be considered an improvement over the more involved laboratory processes used to obtain Peripheral Blood Mononuclear Cells (PBMC) for in vitro culture or over whole blood stimulation assays. Compared to PBMC assays, the TruCulture® accurately represents responsiveness to immune stimuli by human whole blood immune cells as it maintains in its mix all type of immune cells present in the blood, including granulocytes and platelets. Moreover, it is not dependent on cryopreservation as it is often the case with PBMC assays. Furthermore, by relying on a self-enclosed tube is not susceptible to contamination during handling. The available experimental data provided by the manufacturer indicate that the number of cytokines and their secreted levels are similar between TruCulture® and PBMC assays [3]. Compared to whole blood stimulation assays which rely on a relatively short time in culture, the longer incubation periods of the TruCulture® system permits investigation of processes involving both de novo cytokine and chemokine synthesis and for accumulation of mediators for more robust measurements [3].

Accessing whole blood, as described in this approach, may offer a way to identify peripheral biomarkers for patient clinical assessment. It is not taxing for patients as only small blood volumes are required and the ease of handling of the samples is easily integrated with routine clinical sample processing.

## Type of data obtained/readout

Analysis can be based on any technique which detects immunological mediators in culture supernatants, ranging from ELISA to multiplex analysis, to allow for maximal data acquisition. Using TruCulture® can aid in the identification of circulatory biomarkers that may offer information on patient prognosis as important as that provided by the immunoscore at the tumor site [1, 2]. Furthermore characterization of relevant circulatory biomarkers may prove to be critical for non-invasive monitoring disease progression and response to immunotherapeutic treatments.

In healthy volunteers, this technique has been used to successfully quantify circulatory immunological mediators [4–7]. In particular, Mueller and colleagues showed (by ELISA) reproducible subject-specific cytokine reference patterns in healthy individuals, which remained consistent over time [4]. Duffy and colleagues, in an exhaustive study published in Immunity in 2015, were able to differentiate (using Luminex multianalyte profiling) specific inflammatory signatures based on 32 measured mediators for each of the 27 stimulants contained in the TruCulture® tubes they tested with blood from 25 subjects [5].

## Limitations of the approach

For correct interpretation, a negative control tube (i.e. Null) should be included to provide an assessment of the background levels of immunological markers. Whereas the list of off-the-shelf stimulants is increasing, there may be still limitations to their relevance to specific clinical investigations in the oncology field. Given the one-year expiry date on the tubes, it may be that over the course of a clinical trial, a number of tube lots are used. However, equivalence data between different lots can be requested. Tube usage has to be significantly overestimated to account for potential clinical trial issues such as delays in patient recruitment, timing of sampling etc.

## Types of samples needed and special issues pertaining to samples

Proper collection, incubation and storage at −80 °C of samples are critical, but the limited requirement for manipulation provides a significant advantage. The use of a null tube allows for the control of the effects of therapeutic agents present in the whole blood which may influence data interpretation. The usual care applied to supernatant samples needs to be followed including limitation of freeze-thawing cycles and all samples pertaining to a single patients being run at the same time to limit inter-assay variation.

## Level of evidence

The level of evidence for this rather novel technique is currently limited to published studies in healthy individuals. These studies reported the successful quantification of immunological mediators and identification of specific immunological signatures for each of the stimulants used [4–7]. Published papers in patients are limited to first-onset schizophrenia, where analysis of TruCulture® supernatants revealed a distinctive pro-inflammatory signature characterized by altered endothelial cell function and inflammation [8].

There have yet to be any formal published studies using TruCulture® in cancer patients. However, the technology is being increasingly used and reported [7, 9]. For example, we have presented initial data from selected colorectal and pancreatic cancer patients showing that this technique offers a way of reliably obtaining measurable immunological responses in patients before and after therapy with IMM-101, an immunotherapeutic agent based on *Mycobacterium obuense* (NCTC 13365), used in combination with standard chemotherapy or radiation therapy [9]. In contrast, multiplex analysis of patients’ serum samples detects only very low levels of the secreted and systemically circulating cytokines. This is because when using TruCulture®, stimulated immune cells secrete a more varied range of detectable immunological mediators, and levels remains high as they do not become diluted in the serum.

In the future, this approach, characterized by low burden for patients providing samples and ease of use for clinical staff, may become increasingly adopted to monitor patient’s immunological responses so that research in this area may eventually identify prognostic biomarkers to complement those identified at the tumor site.

## Abbreviations

APC: antigen presenting cell
ELISA: enzyme-linked immunosorbent assay
CO_2_: carbon dioxide
